# Autoimmune Complications in Chronic Lymphocytic Leukemia in the Era of Targeted Drugs

**DOI:** 10.3390/cancers12020282

**Published:** 2020-01-23

**Authors:** Candida Vitale, Maria Chiara Montalbano, Chiara Salvetti, Elia Boccellato, Valentina Griggio, Mario Boccadoro, Marta Coscia

**Affiliations:** 1 University Division of Hematology, A.O.U. Città della Salute e della Scienza di Torino, via Genova 3, 10126 Torino, Italy; 2 Department of Molecular Biotechnology and Health Sciences, University of Torino, via Nizza 52, 10126 Torino, Italy

**Keywords:** chronic lymphocytic leukemia, autoimmune hemolytic anemia, immune thrombocytopenia, ibrutinib, idelalisib, venetoclax

## Abstract

Autoimmune phenomena are frequently observed in patients with chronic lymphocytic leukemia (CLL) and are mainly attributable to underlying dysfunctions of the immune system. Autoimmune cytopenias (AIC) affect 4–7% of patients with CLL and mainly consist of autoimmune hemolytic anemia and immune thrombocytopenia. Although less common, non-hematological autoimmune manifestations have also been reported. Treatment of CLL associated AIC should be primarily directed against the autoimmune phenomenon, and CLL specific therapy should be reserved to refractory cases or patients with additional signs of disease progression. New targeted drugs (ibrutinib, idelalisib and venetoclax) recently entered the therapeutic armamentarium of CLL, showing excellent results in terms of efficacy and became an alternative option to standard chemo-immunotherapy for the management of CLL associated AIC. However, the possible role of these drugs in inducing or exacerbating autoimmune phenomena still needs to be elucidated. In this article, we review currently available data concerning autoimmune phenomena in patients with CLL, particularly focusing on patients treated with ibrutinib, idelalisib, or venetoclax, and we discuss the possible role of these agents in the management of AIC.

## 1. Introduction

Chronic lymphocytic leukemia (CLL) represents approximately 20% of all hematological diagnoses, with more than 20,000 new expected cases in the U.S. in 2019 [1]. Diagnosis occurs predominantly in elderly patients, with a median age reported between 67 and 72 years, where men are more frequently affected than women (ratio of 1.7:1) [2].

CLL has a heterogeneous clinical course, which can vary from indolent to, less frequently, aggressive forms. The Rai and Binet clinical staging systems are applied to stratify patients, defining their risk and prognosis. Patients with asymptomatic early stage disease (Rai 0 and Binet A stages) do not require treatment, but should be monitored until disease progression, while therapy should be initiated in patients with advanced disease (Rai III–IV and Binet C stages) or when at least one of the following criteria defining “active disease” is met: (1) progressive bone marrow failure (anemia (hemoglobin (Hb) concentration <11 g/dL for the Rai staging system and <10 g/dL for the Binet staging system) or thrombocytopenia (platelet count <100 × 10⁹/L), or both); (2) massive, or progressive, or symptomatic splenomegaly or lymphadenopathies; (3) progressive lymphocytosis (an increase ≥50% over a two-month period or a lymphocyte doubling time <6 months), in the absence of other contributing factors (e.g., infections, corticosteroid administration); (4) autoimmune complications poorly responsive to corticosteroids; (5) symptomatic or functional extranodal involvement; (6) disease-related symptoms (unintentional weight loss, significant fatigue, or persistent fever without evidence of infection) [3,4]. Different therapeutic options are currently available for patients with CLL, and in recent years, standard chemo-immunotherapy (CIT) has progressively left room to targeted agents such as BTK inhibitors (e.g., ibrutinib), PI3K inhibitors (e.g., idelalisib), and Bcl-2 antagonists (e.g., venetoclax), used alone or in combination with anti-CD20 monoclonal antibodies [5].

It is well known that patients affected by CLL present a profound immune dysregulation, which determines an increased risk of secondary malignancies and opportunistic infections, but also favors the occurrence of autoimmune complications, affecting the course and management of the disease [6,7,8,9,10]. Autoimmune complications have been reported in up to a quarter of CLL patients [11,12]. Among these events, the most significant manifestations are autoimmune cytopenias (AIC), in which autoimmunity preferentially targets blood cells, while non-hematological autoimmunity is undoubtedly less frequent. Notably, it is difficult to determine the exact incidence and prevalence of these events, since their presentation is variable along the course of the disease and in different stages, and diagnostic criteria used in different studies are rather inconsistent. Furthermore, in recent years, the availability of targeted drugs that have dramatically changed the overall prognosis of patients with CLL arguably had an impact on the management of autoimmune phenomena [5].

The aim of this article is to review available data regarding autoimmune phenomena in CLL, with a special focus on the setting of patients treated with currently approved targeted drugs and on the role of these agents in the management of autoimmune complications.

## 2. Pathophysiology and Diagnostic Criteria for Autoimmune Cytopenias in CLL

**Figure 1:**
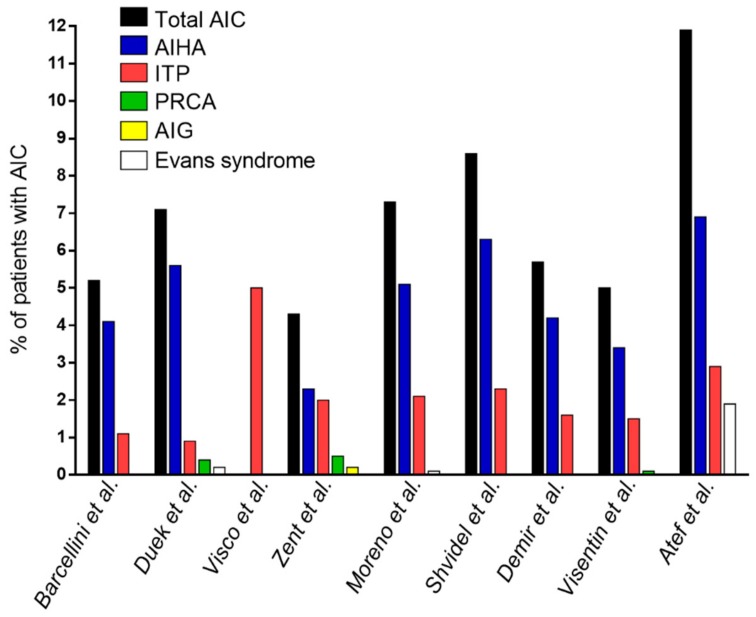
Occurrence of autoimmune cytopenias in patients with CLL. Abbreviations: AIC, autoimmune cytopenia; AIG, autoimmune granulocytopenia; AIHA, autoimmune hemolytic anemia; CLL, chronic lymphocytic leukemia; ITP, immune thrombocytopenia; PRCA, pure red cell aplasia.

The association between AIC and CLL has been described since the late 1960s [13]. Among AIC, autoimmune hemolytic anemia (AIHA) is by far the most common type, followed by immune thrombocytopenia (ITP), and their simultaneous or sequential association is defined as Evans syndrome. Pure red cell aplasia (PRCA) and autoimmune granulocytopenia (AIG) are definitely rarer. Major studies systematically evaluating the occurrence of AIC in patients with CLL are summarized in Table 1 and Figure 1.

### 2.1. Pathophysiology 

The pathophysiology of AIC involves a variety of different immunologic dysfunctions, which have not been completely clarified. The majority of studies focused on the most common forms (i.e., AIHA and ITP), whereas little has been elucidated on the biological mechanisms underlying the development of PRCA and AIG, which are very rare events. 

In the majority of cases, AIC in CLL are mediated by humoral immune mechanisms and in particular by polyclonal high affinity Immunoglobulin G (IgG) auto-antibodies produced by clones of non-malignant B cells and directed against membrane antigens expressed on red blood cells, granulocytes, or platelets [23,24,25]. More rarely, auto-antibodies can also be produced by malignant B cell clones: in this case, they are usually of the Immunoglobulin M (IgM) class and represent a small proportion of the total immunoglobulins detected in patients’ serum [26]. Besides targeting mature blood cells, auto-antibodies can also interfere with erythroblasts or megakaryocytes maturation in the bone marrow, and in this case, they are directed towards precursor cells in various stages of differentiation, preventing proper lineage maturation [23]. 

Several studies have highlighted the concurrent role of cellular immune mechanisms that can sustain AIC in patients with CLL, and T cells are considered the main players in this scenario. Indeed, in these patients, tumor lymphocytes and T cells express altered patterns of surface molecules, impairing their correct interactions during immune responses. This condition seems to be sharpened and supported by an imbalanced cytokine environment. In particular, increased concentrations of IL-10, IL-6, IL-4, and many others cytokines with an immunomodulatory role have been observed in CLL patients and were reported to play a role in AIHA [27,28]. Tumoral B cells themselves can promote AIC by acting as antigen presenting cells and inducing the formation of auto-reactive T cells [29,30]. Imbalanced T cell subsets skewing toward a T helper (Th)2 immune response (a common alteration in cancer, autoimmunity, and infections) has been described in patients with CLL related AIHA [27], and may contribute to the development of AIC. In addition, the expansion of T regulatory cells (Tregs) observed in CLL patients could be implicated in a reduced anti-tumor immune response and in a compromised immunosurveillance, also involving autoimmune disorders [31,32]. 

Reports specifically focusing on the pathophysiology of PRCA described how soluble factors released by T cells or natural killer (NK) cells, such as pro-apoptotic cytokines, selectively impair erythroid precursors colonies in the bone marrow. In this context, the role of T cells is also supported by the common association of PRCA with T large granular lymphocytes (LGL) expansion [33,34].

As a further mechanism, different authors have reported a modified expression of the Toll-like receptor (TLR) pattern in CLL patients. It is well known that TLR are main regulators of innate immunity, being also involved in autoimmune processes [35]. The decrease of TLR2 and TLR4 genes’ expression, as well as the higher expression of TLR9 are widely described in CLL patients [35,36,37]. Consistently, Barcellini et al. have highlighted the role of TLR4 and TLR9 expression in the occurrence of infections, autoimmunity, and disease progression in patients with CLL [38].

### 2.2. Autoimmune Hemolytic Anemia

The most frequently applied definition of AIHA considers the presence of all the following criteria [39,40]:

- Hb levels lower than or equal to 11 g/dL, in the absence of any cytotoxic treatment in the preceding month or other etiology identified;
- evidence of an underlying autoimmune mechanism, such as a positive direct antiglobulin test (DAT) for either IgG or C3 or the presence of cold agglutinin, after exclusion of alternatives (i.e., delayed hemolytic transfusion reaction);
- presence of one or more laboratory marker of hemolysis (high reticulocyte count, low serum haptoglobin levels, increased serum lactate dehydrogenase, or bilirubin levels).

Like idiopathic AIHA, CLL associated AIHA comprises the more frequent warm type, generally due to IgG optimally binding erythrocytes at 37 °C in vitro, and a cold type (cold hemagglutinin disease (CHAD)), usually caused by IgM optimally binding erythrocytes at 4 °C. The results of the DAT test help in discriminating between the two forms, although, in a minority of cases, AIHA can also occur with mixed forms [39].

In the scenery of CLL, the diagnosis of AIHA may be difficult mainly because blood cell count and laboratory values (e.g., LDH level) can be affected by disease progression or concomitant treatment. For this reason, a CT scan or alternative imaging techniques should be performed to evaluate the possible presence of massive or progressive splenomegaly or lymphadenopathies. Furthermore, concomitant causes such as an acute phase response can elevate haptoglobin levels, normally reduced during hemolysis, and reticulocytosis may be absent in the case of bone marrow infiltration. Finally, cytotoxic therapies can increase bilirubin levels and inhibit reticulocyte production. The distinction between AIHA and other etiologies possibly causing anemia, such as occult bleeding, iron or vitamin deficiency, inflammatory conditions, renal dysfunction, and bone marrow failure in patients with progression of disease, is also fundamental. In addition, there are reports of DAT negative AIHA in CLL patients, where the diagnosis has been made by exclusion, in the presence of two or more hemolysis markers [18]. On the contrary, the presence of a positive DAT may not be accompanied by hemolysis and does not constitute *per se* an indication of AIHA diagnosis. 

### 2.3. Immune Thrombocytopenia

The diagnosis of ITP is generally made in the presence of all the listed conditions [16,18,41]:

- otherwise unexplained and sudden fall in platelet count (<100 × 10^9^/L), in the presence of normal bone marrow function (normal or increased number of megakaryocytes at bone marrow examination);
- no evidence of splenomegaly and no cytotoxic treatments within the last month;
- exclusion of other possible causes of thrombocytopenia (e.g., drug induced thrombocytopenia, infections, thrombotic thrombocytopenic purpura, disseminated intravascular coagulation).

The diagnosis of ITP may be cumbersome in patients with concomitant CLL, mainly because thrombocytopenia may manifest as a consequence of bone marrow infiltration by leukemic cells, and the use of the anti-platelet antibody test is not justified due to insufficient sensitivity and specificity [16,17,42,43]. In the diagnostic work-up, a review of peripheral blood smear and bone marrow evaluation could be helpful to correctly identify ITP in patients with CLL. Furthermore, a disease staging including CT scan or other imaging techniques should be considered to detect concomitant CLL progression.

### 2.4. Pure Red Cell Aplasia

The diagnosis of PRCA can be formulated in the presence of the following criteria:

- Hb levels lower than or equal to 11 g/dL, in the absence of hemolysis;
- absolute reticulocytopenia, in the absence of thrombocytopenia or neutropenia;
- exclusion of other causes of red cell aplasia, such as viral infections (e.g., parvovirus B19 or cytomegalovirus) and thymoma.

These features can distinguish CLL associated PRCA from the more common AIHA and from red cell aplasia associated with other diseases [41,44]. From the diagnostic standpoint, a bone marrow examination is needed to exclude that anemia is related to leukemic bone marrow involvement. However, in the presence of massive infiltration of the bone marrow by leukemic cells, PRCA cannot be conclusively excluded. 

### 2.5. Autoimmune Granulocytopenia

A diagnosis of AIG should be considered in the case of:

- persistent neutropenia <0.5 × 10^9^/L in the absence of cytotoxic treatments in the preceding eight weeks;
- absence of granulocyte precursors in the bone marrow.

Secondary AIG usually presents in the setting of systemic autoimmune diseases, particularly systemic lupus erythematous and rheumatoid arthritis, but it is also seen in other clinical situations such as infectious diseases and solid and hematological neoplasms [45]. AIG is a rare occurrence in CLL patients, who typically present serious neutropenic infections [17]. CLL associated AIG is generally considered a diagnosis of exclusion, following the detection of an isolated, persistent, and not otherwise explained neutropenia. In the diagnostic work-up, it is primarily necessary to exclude neutropenia due to bone marrow infiltration from CLL cells, myelodysplastic alterations, or long-term toxicity from previous treatment, including both chemotherapy and anti-CD20 monoclonal antibodies. Of note, rituximab can cause late-onset neutropenia occurring even four or more weeks after the last treatment [46]. Lastly, the presence of a clone of T-LGL, which frequently coexists with CLL and other B cell lymphoproliferative disorders, is also a common cause of AIG [45,47]. With the aim of overcoming the diagnostic challenge, different methods to detect the presence of anti-neutrophil auto-antibodies have been developed, but their specificity and sensitivity are not clearly established in the setting of CLL [48,49]. 

## 3. Non-Hematological Autoimmune Complications in CLL

Different studies described the occurrence of non-hematological autoimmune events in patients with CLL (Table 2). Overall, the most frequent are cases of bullous pemphigus, Hashimoto’s thyroiditis, rheumatoid arthritis, vasculitis, and acquired angioedema, but cases of autoimmune disorders that are extremely rare in the general population have also been reported. High rates of positivity for serological markers of autoimmunity, such as antinuclear antibodies, rheumatoid factor, anti-thyroperoxidase antibodies, and anti-thyroglobulin antibodies, have been described in patients with CLL, also in the absence of clinical autoimmune manifestations [14,20]. Interestingly, non-hematological autoimmune complications are mostly observed in CLL patients with an initial stage of disease [14,20]. 

Due to their rarity and extreme variability, these autoimmune events are difficult to study in a comprehensive manner. Nevertheless, even though there is no strong evidence of a clear predisposition to autoimmunity in CLL patients, the association between CLL and non-hematological autoimmune phenomena is undeniable and is also supported by the described frequent co-occurrence of CLL progression and autoimmune disorder flares [50]. On the other hand, a predisposition to develop CLL in patients with underlying autoimmune disease was not confirmed in two different population-based studies [52,53].

## 4. Prognostic Impact of Autoimmune Complications in CLL

Since the Rai and Binet staging systems do not distinguish between AIC and bone marrow infiltration as the origin of cytopenias, at least a proportion of cytopenias occurring in advanced stage CLL may consist of AIC. It has been shown that patients with anemia or thrombocytopenia due to bone marrow infiltration have a shorter survival compared to patients with cytopenias of autoimmune origin [18,54,55]. Furthermore, opposite to non-hematologic autoimmune phenomena, a higher occurrence of AIC has been observed in patients with advanced clinical stage CLL, thus suggesting heterogeneous pathogenic mechanisms underlying hematologic and non-hematologic autoimmune events [14,18,20,21]. 

Besides clinical staging, additional biomarkers such as ZAP-70 and CD38 expression, serum β2 microglobulin level, immunoglobulin heavy chain variable region genes (IGHV) mutational status, chromosomal aberrations [e.g., del(17p), del(11q)], and gene mutations (e.g., TP53) provide prognostic information in CLL [3]. Several studies presented data on a significant association, in CLL patients, between AIC or other autoimmune disorders and unfavorable prognostic parameters, particularly high lymphocyte count, high β2 microglobulin level, and increased expressions of CD38 and ZAP-70 [15,16,18,21,22,56]. A higher prevalence of unmutated IGHV mutational status was also reported in patients who presented AIHA and ITP secondary to CLL [16,21,57], although this association was not confirmed in all cohorts [18]. The discordance among different reports is arguably dependent on the heterogeneity of the studies and their retrospective nature.

Although several studies reported an association between autoimmune complications and adverse prognostic factors [17,18,21,22,54,55,58], only a minority showed an impact of autoimmune phenomena on overall survival. Shvidel et al. described in their cohort a significantly shorter survival from the time of CLL diagnosis for patients with AIHA compared to patients without AIHA [19]. Accordingly, Visco et al. reported that patients developing AIHA early after CLL diagnosis had a significantly inferior overall survival compared to patients with late-onset or no AIHA occurrence [57]. In their cohort of previously untreated CLL patients, Dearden et al. showed that DAT positivity was predictive of a shorter progression-free and overall survival and that the occurrence of an overt AIHA associated with lower response rates and poorer overall survival [56]. Lastly, ITP was identified as a predictor of poor overall survival, independently of other common clinical prognostic factors [16]. Again, the lack of consistency among data presented in different studies can be mainly attributed to the diverse patient populations, also being influenced by the heterogeneity of criteria used to define AIC.

## 5. Treatment of Autoimmune Complications in CLL 

When additional criteria for CLL treatment are not fulfilled, CLL associated AIC is initially managed with steroids, alone or in association with anti-CD20 monoclonal antibody rituximab, and/or immunosuppressive therapy or, in the case of ITP, with a thrombopoietin (TPO) receptor agonist [3]. In the absence of specific guidelines, the therapeutic approach is generally experience-based and usually mimics the treatment of primary AIC [39,59,60,61]. Of note, the majority of available recommendations refer to the treatment of AIC, whereas specific indications on the management of less frequent non-hematological autoimmune complications do not exist.

### 5.1. Standard Therapy for Autoimmune Complications in CLL

When AIC directed therapies are not sufficient to control autoimmune manifestations, a CLL directed treatment is recommended. Despite the high prevalence of patients with CLL presenting with AIC in the clinical practice, data specifically describing the effects of standard CIT on CLL associated AIC are not conclusive. 

The combination of bendamustine and rituximab (BR) was found to be effective in warm AIHA (*n* = 26), showing an overall response rate of 81% and a time to next treatment of 28.3 months [62]. BR has also been proposed as a therapy in a cohort of 45 patients presenting with chronic CHAD and showed an overall response rate of 71%, including 40% complete responses [63]. 

CIT regimens not typically used for the treatment of patients with CLL have also been evaluated in this setting. The combination of rituximab, cyclophosphamide, and dexamethasone (RCD) was evaluated in 21 patients with CLL associated AIC (mainly AIHA) and showed optimal efficacy (overall response rate 100%), with good tolerability [64]. These data were confirmed in a retrospective study showing, in a larger cohort of refractory CLL associated AIC (*n* = 48) treated with two different RCD regimens, a response in almost 90% of patients and a median duration of response of 24 months [65].

Similar results were achieved in 20 patients with relapsed CLL associated AIC treated with rituximab, cyclophosphamide, vincristine, and prednisone (R-CVP): the overall response rate was 95%, with 70% complete responses and a median duration of response of 21.7 months [66]. 

### 5.2. Targeted Agents for the Treatment of Autoimmune Complications in CLL 

Overall, most evidence supports the efficacy of ibrutinib in treating or at least improving AIC. Cases of successful treatment of AIC both in standard and in high risk patients with refractory AIHA have been reported [67,68,69,70]. Rogers et al. reviewed medical records of 301 patients who received ibrutinib in four clinical trials [71]. In 21% of patients, ibrutinib was given in combination with ofatumumab. Seventy-eight patients (28%) had a history of AIC, and among them, 22 were receiving a concurrent AIC treatment at the time of ibrutinib initiation (14 patients prednisone alone, 3 prednisone plus cyclosporine, 2 romiplostim, 2 cyclosporine, and 1 rituximab). Of note, all cases of AIC were deemed to be controlled at ibrutinib start, and 86% of patients were able to discontinue AIC directed therapy after a median time of 4.7 months.

In an ad hoc analysis of the phase III RESONATE study, which compared ibrutinib versus ofatumumab in previously treated CLL, 38 out of 195 patients treated with ibrutinib (19.5%) had a history of AIC [72]. At ibrutinib start, 21 patients (10.8%) had an ongoing AIHA, with five patients requiring concomitant corticosteroids administration, and 12 patients (6.2%) had an ongoing ITP. Median Hb level and platelet counts improved early after starting ibrutinib and were generally maintained throughout a median follow-up of 18.9 months. Of note, one patient, who was receiving concomitant corticosteroids, was able to discontinue them after 42 days of ibrutinib treatment.

Hampel et al. retrospectively evaluated 193 patients treated with ibrutinib according to clinical practice at the Mayo Clinic [73]. Twenty-nine patients (15%) had a history of previous AIC (11 AIHA, 8 ITP, 5 AIHA and ITP, 3 PRCA, 1 AIG, and 1 aplastic anemia), including 12 patients who were receiving AIC directed therapy at the time of ibrutinib start. Ibrutinib treatment allowed three patients to discontinue AIC directed therapy, after a median time on therapy of two months (range, 0–25 months), and five patients to reduce the intensity of AIC directed therapy. Overall survival and event-free survival from time of ibrutinib start were not significantly different between patients with and without a history of AIC.

Recently, Quinquenel et al. analyzed the outcome of CLL patients from 15 French centers who had an AIC and were treated with ibrutinib or idelalisib [74]. In the ibrutinib cohort (*n* = 25), diagnosis of AIC was AIHA for 16 patients (64%), ITP for 5 patients (20%), Evans syndrome for 3 patients (12%), and PRCA for one patient (4%). Most patients presented with adverse prognostic factors (del(11q), or del(17p), or unmutated IGHV) and had received prior treatment for either CLL or AIC. Overall, the AIC response rate to ibrutinib was 92%. At the time of ibrutinib initiation, 14 patients (72%) were receiving concomitant corticosteroids and three patients (12%) were receiving a TPO receptor agonist. Twelve/fourteen patients who were receiving corticosteroids and 2/3 patients who were receiving TPO receptor agonist were able to discontinue AIC directed therapy, at a median time of three and 12.3 months, respectively.

To date, very little is known regarding the impact of novel BTK inhibitors, such as acalabrutinib, on autoimmune complications in CLL. The recently updated results of a phase II study evaluating acalabrutinib monotherapy in 134 relapsed/refractory CLL showed that among 11 patients with prior AIC, only one had an AIHA recurrence during treatment [75].

Although some authors prefer to avoid idelalisib for the treatment of CLL in the presence of AIC, due to the high incidence of immune driven complications reported with this drug [76], few data on AIC management with idelalisib are available. In the abovementioned study from Quinquenel et al. [74], 19 patients with CLL and AIC (12 AIHA, 6 ITP, 1 Evans syndrome) were treated with idelalisib and rituximab. Interestingly, the AIC response rate to idelalisib was 95%, and eight out of 12 patients receiving corticosteroids were able to discontinue AIC directed treatment. Idelalisib failed to control AIHA only in one case, and all AIHA relapses (*n* = 5) occurred after idelalisib discontinuation.

Data on venetoclax effects on CLL related AIC are so far anecdotal. A CLL patient with del(17p) and refractory AIHA was successfully treated with venetoclax obtaining a response after three months of therapy [77]. Consistently, venetoclax administration was reported to induce an early occurring (i.e., during the rump-up phase) and persistent response in two patients with CLL and concomitant AIC (one ITP and one Evans syndrome) [78].

## 6. Drug-Induced Autoimmune Complications in CLL 

### 6.1. Historical Data on Drug-Induced Autoimmune Complications in CLL 

Besides the therapeutic efficacy on autoimmune complications, different CLL directed drugs have also been reported to trigger AIC, although the mechanisms underlining this phenomenon are not completely understood. It has been hypothesized that the role of chemotherapy in precipitating AIC could depend on a therapy related impairment of Tregs and on an inversion of the CD4:CD8 ratio [79,80]. 

Chemotherapy related AIC occurs with relatively high frequency during treatment with purine analogues. Prospective and retrospective studies including CLL patients treated with single agent fludarabine reported a significant incidence of AIHA, ranging from 11% to 23% [56,80,81]. Only a minority of patients in these studies had a history of AIC preceding the start of treatment. Additionally, a recrudescence of AIHA was experienced by the majority of patients undergoing a re-challenge of fludarabine after an initial control of the hemolysis, thus suggesting a direct role of this drug in the onset of the autoimmune phenomenon [56,81,82]. Although less frequently, treatment emergent ITP has also been reported in the setting of patients with lymphoproliferative diseases receiving a fludarabine containing regimen [79]. Within purine-analogues, also cladribine has been associated with the development of AIC, as reported in a retrospective study showing treatment emergent AIHA in 22% of treated patients [83]. Cases of treatment induced AIC have also been reported in CLL patients treated with other agents, such as pentostatin, bendamustine, chlorambucil, and alemtuzumab [56,84,85,86,87], thus supporting the hypothesis of an association of autoimmune manifestations with the underlying disease more than the responsibility of any specific drug.

Interestingly, the addition of other agents, such as cyclophosphamide and rituximab, to fludarabine seems to reduce the risk of autoimmune complications. In the prospective multicenter LRF CLL4 trial, more than 700 patients were randomly assigned to receive chlorambucil, fludarabine, or fludarabine plus cyclophosphamide (FC), with an AIHA incidence of 12%, 11%, and 5% in the three arms, respectively (*p* = 0.01) [56]. Consistently, in a study including 300 patients treated with the association of FC with rituximab (FCR), the rate of therapy induced AIC was only 6.5% (17 cases of AIHA, two cases of PRCA) [88]. 

Non-hematological autoimmunity related to chemotherapy is a rare event. Only sporadic cases have been reported, all in association with purine analogues. Skin manifestations, such as erythema anulare, vasculitis, pemphigus, and toxic epidermal necrolysis have been described [89,90], as well as p-ANCA positive glomerulonephritis [91]. Paradoxical flares of pre-existent autoimmune diseases (a case of polyarthritis and one of lupic glomerulonephritis) soon after fludarabine start were also reported [90].

### 6.2. Drug Induced Autoimmune Complications in the Era of Targeted Agents for CLL Treatment 

#### 6.2.1. Ibrutinib

Cases of treatment emergent AIC arising mainly in the first weeks of treatment with ibrutinib have been reported. In two case reports, AIHA developed soon after ibrutinib start: patients withheld ibrutinib and started steroids until resolution of AIHA, and were able in both cases to re-challenge successfully ibrutinib without any evidence of AIC recurrence [92,93]. In another report, nine of 13 patients with CLL and signs of AIC treated with ibrutinib showed a temporary worsening of AIC during the first weeks of therapy [94]. Interestingly, this phenomenon was observed also in three patients who were receiving ibrutinib in combination with rituximab. In the majority of patients, the AIC flare was resolved or controlled without ibrutinib discontinuation and with temporary addition of steroids or other immunosuppressive therapies. 

**Figure 2:**
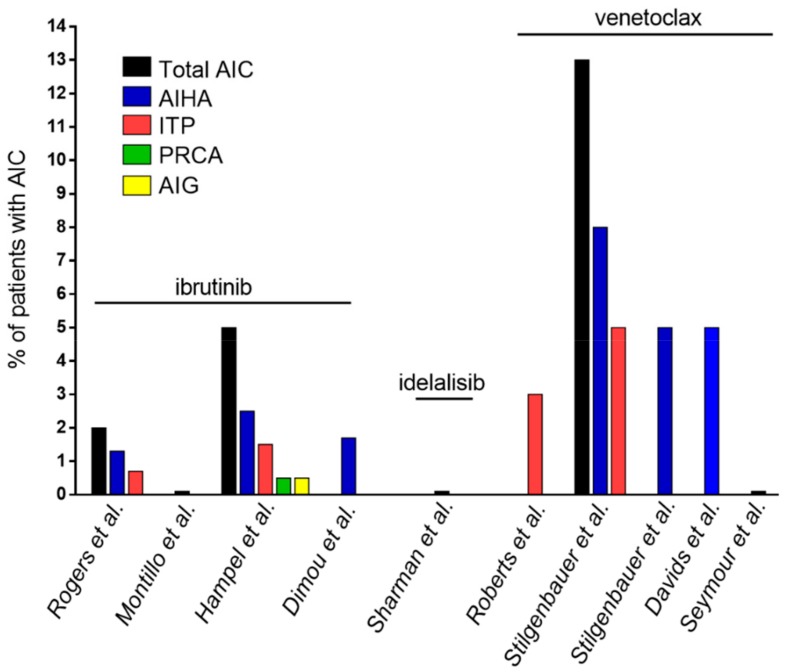
Occurrence of autoimmune cytopenias in patients with CLL treated with targeted agents. Abbreviations: AIC, autoimmune cytopenia; AIG, autoimmune granulocytopenia; AIHA, autoimmune hemolytic anemia; CLL, chronic lymphocytic leukemia; ITP, immune thrombocytopenia; PRCA, pure red cell aplasia.

More systematic evaluations have shown that the overall incidence of treatment emergent AIC in patients receiving ibrutinib is generally low (Table 3 and Figure 2). In a monocentric retrospective study by Rogers et al., only six out of 301 patients treated with ibrutinib in the setting of clinical trials experienced a treatment emergent AIC [71]. In the phase III RESONATE study comparing ibrutinib versus ofatumumab in relapsed or refractory CLL, after a median treatment duration of 18.3 months, no new cases of AIC occurred in the ibrutinib arm, and ibrutinib did not seem to trigger AIC recurrence in patients with a previous history of AIC [72]. Moreover, in a real-life analysis conducted on 193 patients with CLL treated with ibrutinib in routine clinical practice, Hampel et al. observed treatment emergent AIC in 6% of patients, consisting of 5 cases of AIHA, 3 ITP, 1 PRCA, 1 aplastic anemia, and 1 AIG [73]. The time to onset of treatment emergent AIC was quite variable, ranging from six to 319 days following ibrutinib initiation (median time, 59 days). Of note, treatment emergent AIC was seen exclusively in patients with unmutated IGHV and was associated with a shorter event-free survival. Lastly, a retrospective analysis conducted on a cohort of 58 patients treated in a single center with ibrutinib outside of clinical trials, including nine patients with a prior history of AIC, showed in only one patient a recurrence of AIHA, which happened after six months and resolved by adding steroids and cyclosporine, thus allowing ibrutinib re-challenge [95].

Non-hematological autoimmune events related to ibrutinib are mostly anecdotal. Four cases of suspected pneumonitis, with no evidence of infective causes and responding to ibrutinib withdrawal and steroid treatment have been described [102]. A case of autoimmune myelitis after two months of ibrutinib treatment was reported [103]. The patient continued ibrutinib, and the acute event was managed by adding methylprednisolone, with a complete resolution of neurologic symptoms after 10 months and no subsequent recurrence. Another ibrutinib treated patient developed palindromic rheumatoid arthritis, which was managed through ibrutinib dose reduction and addition of immunosuppressive therapy [104].

#### 6.2.2. Idelalisib 

To our knowledge, there are no published data specifically describing the frequency of treatment emergent AIC during idelalisib and idelalisib based regimens, thus suggesting a low frequency of the phenomenon (Table 3 and Figure 2). This assumption is further supported by the results of a large phase III study of idelalisib and rituximab in relapsed or refractory CLL, which did not mention AIC among the most frequent adverse events (AEs), not even in the recent long-term update [96,105]. 

In contrast, idelalisib is well known for its non-hematological autoimmune effects: autoimmune pneumonitis, transaminitis, and colitis are indeed peculiar AEs related to this drug. In a phase II trial that enrolled 64 treatment-naïve elderly patients with CLL, transaminitis was reported in 67% of patients, being of grade ≥3 in 23%. Diarrhea or colitis occurred in 64% of patients and was of grade ≥3 in 42%, whereas pneumonitis was less frequent (reported in 3% of patients) [106]. In the already mentioned phase III pivotal trial evaluating patients with relapsed/refractory CLL, a prolonged exposure to idelalisib induced diarrhea in 46.4% of patients (16.4% grade ≥3), colitis in 10.9% of patients (8.2% grade ≥3), and pneumonitis in 10% of patients (6.4% grade ≥3) [96]. In contrast to the previously mentioned AEs, whose incidence increased with longer drug exposure, the rate of hepatic toxicity remained stable compared to the shorter follow-up (AST elevation all-grade 28.2%, grade ≥3 5.5%; AST elevation all-grade 39.1%, grade ≥ 39.1%) [96,105].

From a mechanistic standpoint, preclinical evidence supports the hypothesis of a specific on-target off-tumor effect, since mice with a genetic inactivation of PI3Kδ develop autoimmune colitis [107]. The autoimmune etiology of these phenomena, with a special interest in hepatotoxicity, has also been investigated by Lampson et al., who enrolled 24 treatment-naïve patients in a clinical trial with idelalisib in combination with ofatumumab [108]. The authors reported all-grade transaminitis in 19/24 patients (13/19 grade ≥3). Patients who developed transaminitis had a significantly lower number of circulating Tregs and increased serum levels of pro-inflammatory cytokines. Additionally, an infiltrate of activated CD8+ T cells was found in liver biopsy specimens from two patients with transaminitis. The increased frequency of liver toxicity observed in younger and previously untreated patients supports the hypothesis of a main pathogenetic role played by a more preserved immune system, which is capable of inducing stronger autoimmune reactions. Due to the peculiarity of idelalisib related toxicities, management recommendation have been published to minimize the severity of the AEs, focusing on the importance of the early recognitions of warning symptoms, the prompt addition of steroids, and the temporary suspension of idelalisib [109,110]. 

#### 6.2.3. Venetoclax

Although data on venetoclax are more limited, due to the smaller number and shorter follow-up of patients treated to date, treatment emergent AIC have been described (Table 3 and Figure 2). The phase I study of venetoclax in relapsed or refractory CLL (dose escalation cohort + expansion cohort, *n* = 116) reported among serious AEs a 3% incidence of ITP, while no AIHA occurred [97]. In the phase II pivotal trial, which enrolled relapsed/refractory CLL patients with del(17p) (*n* = 107), 8% of patients developed AIHA (grade ≥3 7%) and 5% developed ITP (all of grade ≥3) [98]. A following update evaluating the outcome of 158 patients, at a median time on venetoclax of almost two years, showed a 5% incidence of serious AIHA [99]. Of note, for this trial, the presence of an uncontrolled AIC at the time of enrollment was one of the exclusion criteria. 

A pooled analysis of three phase I and II trials (*n* = 350), including the two abovementioned studies, reported 17 cases of treatment emergent AIHA (5%), 14 of whom (4%) were graded ≥3 [100]. A total of 10 cases were deemed to be serious AEs, including three patients with documented pre-existing AIHA. The events occurred at a median time from venetoclax start of 2.6 months (range 0.07–12.4 months). AIHA led to a venetoclax dose reduction in 2 patients, interruption in 4 patients, and discontinuation in 2 patients. While the overall incidence of ITP in the cohort was not reported, it was reported as the reason for venetoclax interruption in four patients. 

In the phase III Murano trial, among 194 relapsed/refractory patients with CLL randomized to receive venetoclax in combination with rituximab, two patients discontinued treatment for AIHA and one for ITP (vs. none in the BR control arm), but the overall incidence of AIC was not reported, possibly due to the low rate [101]. 

Of interest, none of the cited studies reported the occurrence of non-hematological autoimmune complications.

## 7. Conclusions and Perspectives

In patients with CLL, AIC are a common occurrence, ranging from 4 to 7%. The variability of incidence reported among different studies reflects the heterogeneity of the analyzed cohorts, but also the diagnostic challenges and the inconsistency of the criteria that were used to define AIC. Non-hematological autoimmune manifestations are less frequent and encompass a wide range of different clinical disorders, rendering even more arduous a precise characterization of their incidence, correlation with CLL, and prognostic impact. Collectively, data suggest that AIC tend to associate with an advanced stage of disease and adverse prognostic factors in CLL, but an impact on overall survival has not been definitively demonstrated.

Due to the recent introduction of targeted agents (i.e., ibrutinib, idelalisib, and venetoclax) in the treatment armamentarium for CLL, it is of interest to assess the role of these molecules in the context of autoimmune complications, considering both their therapeutic function and their possible influence in eliciting autoimmune phenomena. Of note, the activity of targeted drugs in the treatment of CLL associated AIC has not been comprehensively evaluated to date, also due to exclusion of patients with active AIC from the pivotal clinical trials and to the absence of studies directly investigating the role of these novel signal inhibitors in this setting. Furthermore, no guidelines are available to direct the management of patients who develop AIC during the treatment with targeted drugs. Currently, data are progressively emerging, since ibrutinib, idelalisib, and venetoclax are used to treat a growing number of patients with CLL, including those with a previous history of AIC and patients with active AIC whose autoimmune manifestations could not be otherwise controlled.

Currently available data show that, despite the reported occurrence of AIC flares in the early phase of treatment, ibrutinib appears to determine an improvement in the majority of patients with pre-existing AIC and to induce a low rate of treatment emergent AIC. Idelalisib is mainly responsible for the triggering of non-hematological autoimmune complications, whereas data on its use in the management of AIC are limited. During venetoclax therapy, treatment emergent AIC were also reported, but the role of this drug in the treatment of autoimmune complications of CLL is not yet well defined. Furthermore, the beneficial effect of co-administering an anti-CD20 monoclonal antibody with a targeted drug to gain a better control of pre-existing AIC and prevent treatment emergent events still needs to be clarified.

The therapeutic efficacy of targeted drugs on CLL associated AIC may be arguably a consequence of the control achieved over the underlying CLL by these highly effective drugs. However, it is also important to highlight that, besides their direct anti-neoplastic effect, these molecules exert complex activities on the host immune system, which may potentially be responsible for either the control or the trigger of autoimmune phenomena. 

BTK inhibition with ibrutinib does not only affect tumor B cells, but can have a multifaceted effect on different components of the immune system [111]. Based on its demonstrated influence on the T cell and monocyte/macrophage compartments, ibrutinib might have a role in controlling autoimmunity [112,113,114]. From the molecular standpoint, ibrutinib is also an irreversible inhibitor of interleukin-2 inducible kinase (ITK), driving a Th1 skewed profile in CD4+ T cell populations isolated from CLL patients [113]. Similarly, the molecular targets of idelalisib are not restricted to the B cell compartment, and the inhibition of PI3Kδ decreases the production of various inflammatory and anti-apoptotic cytokines by T cells and counteracts the infiltrative capacity of macrophages both at the tumor site and within the target tissues of autoimmune reactions [115,116,117,118,119]. The inhibitory activity of idelalisib on the T cell component, and in particular on Tregs, has been implicated in the pathogenesis of idelalisib induced non-hematological autoimmune side effects [108,120]. Venetoclax might exert a control on the apoptotic pathways also in non-neoplastic cells: Bcl-2 associated dysregulation of lymphocyte apoptosis can contribute to the pathogenesis of autoimmune diseases, and ABT-737, a potent inhibitor of Bcl-2, Bcl-xL, and Bcl-w, significantly reduces disease severity in tissue specific and systemic animal models of autoimmunity [121]. More recently, it has been shown that treatment of patients with CLL with venetoclax can impact non-neoplastic immune cells [122], and the immune recovery that follows the elimination of leukemic cells might be implicated in the occurrence of autoimmune events.

The future clinical application of adoptive cell therapies in CLL, such as chimeric antigen receptor modified T cells (CAR T cells), which are demonstrating a promising role in the treatment of B cell malignancies [123], will need to take into account the importance of autoimmune complications in this setting. As for initial studies evaluating targeted agents, to date, most CAR T cell trials have excluded patients with active autoimmune manifestations, and the impact of this adoptive immunotherapy strategy on autoimmune complications still needs to be clarified. Both in clinical trials and in the real life setting, it will be of great interest to assess whether anti-CD19 CAR T cell induced B cell aplasia may affect the onset and outcome of autoimmune phenomena. 

Many uncertainties still remain regarding the impact of targeted agents on autoimmune phenomena in CLL, but we can anticipate that a wide range of information coming from translational studies, as well as the increasing availability of long-term follow-up data from clinical trials and the extended use of these drugs in the clinical practice will provide more conclusive data in the near future.

## Figures and Tables

**Table 1:** Main studies describing the occurrence of autoimmune cytopenias in patients with CLL.

Authors	Type of Study	Number of CLL Patients	Type of Cohort	Patients with AIC, *n* (%)
Barcellini et al. [14]	Multicentric, prospective + retrospective	3150	Treatment-naïve + pre-treated patients	Total 164 (5.2%)
				AIHA 129 (4.1%)
				ITP 35 (1.1%)
Duek et al. [15]	Single center, retrospective	964	Treatment-naïve + pre-treated patients	Total 69 (7.1%)
				AIHA 54 (5.6%)
				ITP 9 (0.9%)
				PRCA 4 (0.4%)
				Evans syndrome 2 (0.2%)
Visco et al. [16]	Multicentric, retrospective	1278	Treatment-naïve + pre-treated patients	ITP 64 (5%)
Zent et al. [17]	Single center, retrospective	1750	Treatment-naïve + pre-treated patients	Total 75 (4.3%)
				AIHA 41 (2.3%)
				ITP 35 (2%)
				PRCA 9 (0.5%)
				AIG 3 (0.2%)
				(10 patients more than one type)
Moreno et al. [18]	Single center, retrospective	960	Pre-treated patients	Total 70 (7.3%)
				AIHA 49 (5.1%)
				ITP 20 (2.1%)
				Evans syndrome 1 (0.1%)
Shvidel et al. [19]	Multicentric, retrospective	1477	Treatment-naïve + pre-treated patients	Total 127 (8.6%)
				AIHA 93 (6.3%)
				ITP 34 (2.3%)
				(including 12 cases with Evans syndrome)
Demir et al. [20]	Single center, prospective	192	Treatment-naïve + pre-treated patients	Total 11 (5.7%)
				AIHA 8 (4.2%)
				ITP 3 (1.6%)
Visentin et al. [21]	Single center, retrospective	795	Treatment-naïve + pre-treated patients	Total 40 (5%)
				AIHA 27 (3.4%)
				ITP 12 (1.5%)
				PRCA 1 (0.1%)
Atef et al. [22]	Single center, retrospective	101	NA	Total 12 (11.9%)
				AIHA 7 (6.9%)
				ITP 3 (2.9%)
				Evans syndrome 2 (1.9%)
				(31 patients with combined autoimmune and infiltrative etiology, 30.7%)

Abbreviations: AIC, autoimmune cytopenia; AIG, autoimmune granulocytopenia; AIHA, autoimmune hemolytic anemia; CLL, chronic lymphocytic leukemia; DAT, direct antiglobulin test; ITP, immune thrombocytopenia; NA, not available; PRCA, pure red cell aplasia.

**Table 2:** Reported cases of non-hematological autoimmune phenomena in CLL.

Authors	Barcellini et al. [14]	Duek et al. [15]	Visentin et al. [21]	Demir et al. [20]	Jung et al. [50]	Alattar et al. [51]
	CLL *n* = 3150	CLL *n* = 964	CLL *n* = 795	CLL *n* = 192	6 cases reported	3 cases reported
**Skin immune complications**	*n* = 9	*n* = 8	*n* = 5	*n* = 1	-	-
	(bullous pemphigoid)		(*n* = 3 bullous pemphigoid)	(bullous pemphigoid)		
**Hashimoto’s thyroiditis**	*n* = 8	*n* = 15	*n* = 12	-	*n* = 2	-
**Grave’s disease**	-	*n* = 3	*n* = 4	-	-	-
**Rheumatoid arthritis**	*n* = 4	*n* = 4	*n* = 4	-	*n* = 1	-
**Vasculitis**	*n* = 1	*n* = 5	*n* = 3	-	*n* = 2	-
			(Horton arteritis)			
**Sj** **ӧgren syndrome**	*n* = 1	*n* = 3	*n* = 3	-	-	-
**Systemic lupus erythematosus**	*n* = 1	*n* = 2	-	-	-	-
**Angioneurotic edema**	-	*n* = 2	-	-	*n* = 1	-
**Multiple sclerosis**	-	*n* = 2	-	-	-	-
**Acquired angioedema**	-	-	-	*n* = 3	-	-
**Ulcerative colitis**	*n* = 1	*n* = 1	-	-	-	-
**Acquired von Willebrand disease**	-	-	-	-	*n* = 1	*n* = 3
**Autoimmune gastritis**	*n* = 1	-	-	-	-	-
**Autoimmune hepatitis**	-	-	-	*n* = 1	-	-
**Autoimmune glomerulonephritis**	*n* = 1	-	-	-	-	-
**Autoimmune polyneuropathy**	*n* = 1	-	-	-	-	-
**Raynaud’s disease**	*n* = 1	-	-	-	-	-
**Polymyositis dermatomyositis**	*n* = 1	-	-	-	-	-
**Ankylosing spondylitis**	-	*n* = 1	-	-	-	-
**Pernicious anemia**	-	*n* = 1	-	-	-	-
**Myasthenia gravis**	-	-	-	-	*n* = 1	-

Abbreviations: CLL, chronic lymphocytic leukemia.

**Table 3:** Autoimmune cytopenias in patients with CLL treated with targeted agents.

Authors	Type of Study	Number of CLL Patients	Type of Cohort	Drug	Patients with AIC, *n* (%)
Rogers et al. [71]	Single center, retrospective (patients treated in 4 different clinical trials)	301	Treatment-naïve + pre-treated patients	Ibrutinib ± ofatumumab	Total 6 (2%)
					AIHA 4 (1.3%)
					ITP 2 (0.7%)
Montillo et al. [72]	Multicenter, prospective	195	Pre-treated patients	Ibrutinib	Total 0
Hampel et al. [73]	Single center, retrospective (clinical practice)	193	32 treatment-naïve (17%), 161 pre-treated patients (83%)	Ibrutinib	Total 11 (5%)
					AIHA 5 (2.5%)
					ITP 3 (1.5%)
					PRCA 1 (0.5%)
					AIG 1 (0.5%)
					Aplastic anemia 1 (0.5%)
Dimou et al. [95]	Single center, retrospective (clinical practice)	58	11 treatment-naïve (19%),	Ibrutinib	AIHA 1 (1.7%)
			47 pre-treated patients (81%)		
Sharman et al. [96]	Multicenter, prospective	110	Pre-treated patients	Idelalisib + rituximab	None reported
Roberts et al. [97]	Multicenter, prospective	116	Pre-treated patients	Venetoclax	Among SAEs:
					ITP 2 (3%)
Stilgenbauer et al. [98]	Multicenter, prospective	107	Pre-treated patients	Venetoclax	Total 13 (13%)
					AIHA 8 (8%)
					ITP 5 (5%)
Stilgenbauer et al. [99]	Multicenter, prospective	158	5 treatment-naïve (3%),	Venetoclax	Among SAEs:
			153 pre-treated patients (97%)		AIHA 8 (5%)
Davids et al. [100]	Pooled analysis (3 prospective trials)	350	Pre-treated patients	Venetoclax	AIHA 17 (5%)
Seymour et al. [101]	Multicenter, prospective	193	Pre-treated patients	Venetoclax + rituximab	No grade ≥3 AIC reported

Abbreviations: AIC, autoimmune cytopenia; AIG, autoimmune granulocytopenia; AIHA, autoimmune hemolytic anemia; CLL, chronic lymphocytic leukemia; ITP, immune thrombocytopenia; PRCA, pure red cell aplasia; SAEs, serious adverse events.
